# Community prevalence of borderline personality disorder and associated sociodemographic factors. Systematic review

**DOI:** 10.1192/j.eurpsy.2025.1928

**Published:** 2025-08-26

**Authors:** I. Ramos Suárez, B. G. Martínez, M. G. Jiménez

**Affiliations:** 1 Psychiatry, San Cecilio University Clinical Hospital; 2 Psychiatry, Granada University of Medicine, Granada, Spain

## Abstract

**Introduction:**

Borderline Personality Disorder (BPD) is one of the most prevalent and debilitating personality disorders in mental health. It is characterized by a persistent pattern of instability in interpersonal relationships, self-image, and affect, along with marked impulsivity.

Available epidemiological data suggest that BPD affects approximately 1-2% of the general population. However, variability in diagnostic criteria and assessment methodologies has made it difficult to obtain comparable results across different studies and geographic contexts.

**Objectives:**

This systematic review aims to synthesize current evidence on the epidemiology of BPD, exploring its prevalence and its sociodemographic correlates.

**Methods:**

This systematic review focused on studies examining the prevalence of Borderline Personality Disorder (BPD) in the general population, as well as the associated sociodemographic factors.

Articles that assessed the prevalence of BPD in adult community populations were included. BPD had to be diagnosed according to the DSM-IV, DSM-5, or ICD-10 criteria, and studies needed to use structured interviews or validated questionnaires. The minimum sample size for included studies was 300 participants to ensure the robustness of the data.

Studies focusing on specific populations (such as hospitalized patient groups) and those that did not differentiate between BPD and other personality disorders were excluded.

An extensive search was conducted in the PubMed and MEDLINE databases.

**Results:**

The initial search yielded a total of 325 results: 139 articles from PubMed and 186 from MEDLINE. Finally, 11 articles were included in the final review. The search and selection process is detailed in the provided image.

The prevalence of Borderline Personality Disorder (BPD) in the general population varied considerably among the included studies. Notably, no studies from Africa or Asia met the inclusion criteria for this review, limiting the generalizability of the results on a global scale. Most of the included studies were conducted in European countries and the United States. Overall, the results suggest variability in BPD prevalence rates depending on geographic region and diagnostic methods employed.

Sociodemographic variables such as gender, age, ethnicity, education level, marital status, income level, location of residence, and employment status showed varying degrees of association with Borderline Personality Disorder (BPD).

**Image 1:**

**Figure:**
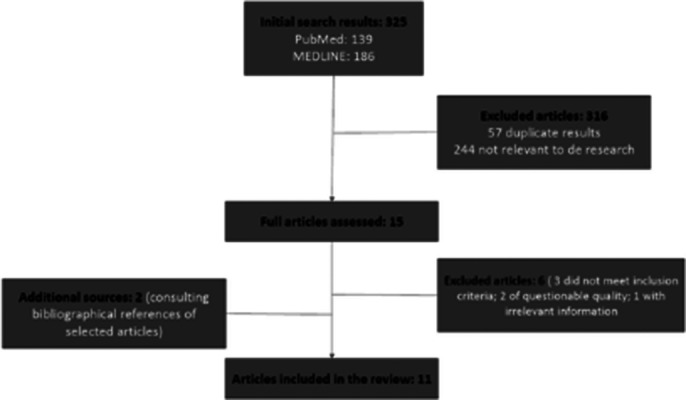

**Conclusions:**

The final conclusions will be included in the full version of the systematic review, once all sociodemographic variables have been thoroughly evaluated. This detailed analysis will also assess the extent of these variables’ involvement, along with how the different diagnostic criteria used may influence the prevalence of Borderline Personality Disorder (BPD) observed in the samples from the various countries included in the study.

**Disclosure of Interest:**

None Declared

